# Protective Effect of Carbon Dots Derived from *Salvia miltiorrhiza* Pretreatment in Acute Myocardial Infarction in Rats

**DOI:** 10.3390/nano15030242

**Published:** 2025-02-05

**Authors:** Liyang Dong, Menghan Li, Tianyou Cao, Yafang Zhao, Shuxian Wang, Peng Zou, Yue Zhang, Huihua Qu, Yan Zhao, Hui Kong

**Affiliations:** 1 School of Traditional Chinese Medicine, Beijing University of Chinese Medicine, Beijing 100029, China; dly1613@163.com (L.D.); lmh0698@163.com (M.L.); zyf2640594023@163.com (Y.Z.); zywsx0803@163.com (S.W.); zoupeng347@163.com (P.Z.); quhuihuadr@163.com (H.Q.); zhaoyandr@163.com (Y.Z.); 2 Shenzhen Institutes of Advanced Technology, Chinese Academy of Sciences, Shenzhen 518055, China; tycao221@163.com; 3 School of Life Science, Beijing University of Chinese Medicine, Beijing 100029, China; 201801024@bucm.edu.cn

**Keywords:** carbon dots, novel nanomedicine, *Salvia miltiorrhiza* Carbonisata, acute myocardial infarction, oxidative stress

## Abstract

Acute myocardial infarction is an ischemic injury of the myocardium caused by an imbalance in the blood supply to myocardial tissues, which poses a serious threat to human life and health. Oxidative stress has been recognized as a significant contributor to acute myocardial infarction. *Salvia miltiorrhiza* Carbonisata (SMC) is among the most frequently employed herbal remedies for the treatment of acute myocardial infarction; however, the exact identity of its principal active constituents is not well defined. Research indicates that carbon dots (CDs) exhibit significant biological properties. Consequently, we initially synthesized carbon dots (CDs) from *Salvia miltiorrhiza* Carbonisata, with the objective of exploring how SMC-CDs mitigate isoproterenol (ISO)-induced myocardial infarction (MI) in rats. The results showed that the pretreatment with SMC-CDs markedly enhanced compromised cardiac function, mitigated myocardial fibrosis and the infiltration of inflammatory cells, decreased the size of the infarct, and suppressed cardiomyocyte apoptosis. Furthermore, the antioxidant properties of myocardial tissue were enhanced, and oxidative stress caused by free radicals was effectively mitigated by SMC-CDs, which succeeded in reducing levels of myocardial enzymes and elevating the activity of relevant ATPases. This implies that SMC-CDs could be a potential candidate for novel nanomedicine strategies designed to address cardiovascular ailments.

## 1. Introduction

Cardiovascular disease (CVD) is one of the leading causes of mortality and impairment worldwide. Acute myocardial infarction (AMI), causing irreversible heart failure, life-threatening arrhythmias, and sudden cardiac death, is the predominant form of CVD morbidity [1]. At present, the incidence of AMI in China is rising year by year, and the trend is young. By 2030, the number of people suffering from this disease may exceed 23 million, posing a substantial threat to people’s lives and health [2]. Patients with AMI tend to have a rapid onset and poor prognosis. The initial manifestation of the disease leads to a dramatic reduction or interruption of coronary blood flow, which results in acute ischemia and hypoxic necrosis of cardiomyocytes in the corresponding blood-supplying areas [3].

Most of the therapeutic strategies for acute myocardial infarction are timely reperfusion of the ischemic area, such as pharmacological thrombolytic therapy and percutaneous coronary intervention, which can improve the symptoms and prognosis of patients with AMI to some extent [4]. However, the restoration of coronary blood flow can cause reperfusion injury to myocardial tissue and eventually lead to further expansion of myocardial infarction area [5]. In Western medicine, conservative therapy involves administering antithrombotic medications, β-blockers, lipid-lowering medications, nitrates, calcium channel blockers, and angiotensin-converting enzyme inhibitors. Nonetheless, the clinical efficacy of these drugs remains unsatisfactory and is associated with a wide range of adverse effects [6]. Consequently, many researchers have focused on the advancement of new medications to prevent and treat the occurrence of ischemic heart disease with minimal side effects. Numerous clinical practices and studies indicate that traditional Chinese herbs possess distinctive advantages in preventing and treating ischemic heart disease. Therefore, the current research focus lies in discovering a safe and efficient traditional Chinese medicine for the prevention and treatment of acute myocardial infarction [7,8].

In recent years, nanotechnology has become a prominent field of study, greatly impacting progress and innovation in the medical domain, particularly in nano-pharmaceuticals and nano-biosensors [9]. As a novel carbon nanomaterial, carbon dots (CDs) exhibit a unique nanoscale dimension of less than 10 nm and are imbued with a diverse range of organic functional groups, which have also been extensively explored across various fields due to their exceptional characteristics, such as excellent biocompatibility, reliable optical properties, and safety [10,11].

The high-temperature carbonization procedure of Chinese herbal charcoal medicine resembles the preparation process of carbon dots in nanomaterials. This observation has prompted us to inquire scientifically whether charcoal medicine also contains nano-like components similar to carbon dots [12]. The team has previously used modern nanotechnology to analyze and study the aqueous solutions of a variety of charcoal drugs, finding nanoparticles with physical and chemical properties similar to carbon dots, such as particle size and structure. It was found that these CDs, beyond their traditional hemostatic effects, also exhibit a range of biological functions, such as anti-ischemia reperfusion injury, anti-anxiety, anti-inflammatory, anti-cold, and anti-gout [13]. These studies not only expand the clinical application of Chinese medicine, but also provide ideas and methods for the research and development of new Chinese medicines.

*Salvia miltiorrhiza* was initially documented in the Shennong Ben Cao Jing (Classic of the Materia Medica of the Divine Husbandman), originating from the desiccated root and rhizome of the plant from the Labiatae family. In the treatment of modern diseases, *Salvia miltiorrhiza* is extensively employed in cardiovascular diseases, such as acute myocardial infarction, myocarditis, arrhythmia, hypertensive heart disease, pulmonary heart disease, and various other conditions [14]. Studies indicate that traditional herbs such as *Scutellaria baicalensis* exhibit significant cardioprotective effects when formulated into nanoparticle delivery systems [15]. Carbon dots made from *Salvia miltiorrhiza*, however, have never been used before. Considering this, the application of CDs for the management and treatment of acute myocardial infarction has become a focal point in contemporary research, meriting further exploration.

In this study, we have created a new type of carbon dots (CDs) using an eco-friendly, one-step calcination method, with *Salvia miltiorrhiza* being the sole source of carbon. Electron microscopy and optical instrumentation were used to analyze the structural features and optical properties of SMC-CDs, concurrently investigating their intricate functional groups and bonding patterns. We performed experimental trials on animals using an isoproterenol (ISO)-induced acute myocardial infarction (AMI) model in rats, investigating the cardioprotective effects of SMC-CDs in this ISO-induced model.

## 2. Results

### 2.1. High-Performance Liquid Chromatography Data Analysis

**Figure 1:**
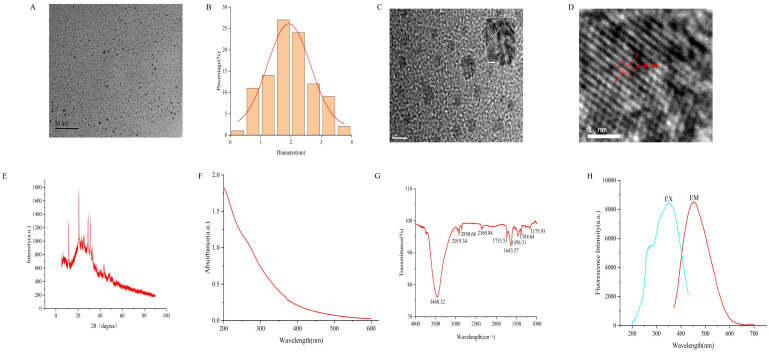
Morphological and optical characterizations of SMC-CDs. (**A**) Transmission electron microscopy (TEM) images of SMC-CDs. (**B**) Particle size distribution histogram of SMC-CDs. (**C**,**D**) High-resolution TEM (HRTEM) images of SMC-CDs. (**E**) X-ray diffraction pattern of SMC-CDs. (**F**) UV-visible spectra of the SMC-CDs. (**G**) Fourier transform infra-red (FTIR) spectra of SMC-CDs. (**H**) Excitation and emission fluorescence spectra of SMC-CDs.

The morphological structure, particle size distribution, lattice spacing and spectral properties of SMC-CDs are shown below. The appearance of SMC-CDs was similar to particles spherical, with uniform distribution and excellent dispersion. Under low-resolution transmission electron microscopy, their external features were distinctly visible, with particle sizes ranging from 0.5 to 3.5 nm, primarily clustered between 1.5 and 2.5 nm, confirming a normal distribution (Figure 1A,B). The lattice spacing of SMC-CDs was observed to be well defined under high-resolution transmission electron microscopy and analyzed by Digital Micrograph to be 0.206 nm (Figure 1C,D). Furthermore, the internal spatial structure of SMC-CDs was further analyzed using XRD. As shown in Figure 1E, a typical amorphous diffraction peak is visible for SMC-CDs with diffraction angle of 2θ = 23.65°. The UV spectra indicate that the presence of conjugated C=O and C=N bonds, along with n-π* electronic transitions in the aromatic sp2 region, resulting in SMC-CD displaying a wide spectrum with a minor absorption peak at approximately 370 nanometers [16]. Based on the FTIR analysis, the SMC-CD shows absorption peaks at 3460.32 cm^−1^, 2919.34 cm^−1^, 2850.68 cm^−1^, 1643.57 cm^−1^, 1384.64 cm^−1^, and 1175.93 cm^−1^. The analysis reveals that the prominent absorption peak at 3460.32 cm^−1^ is attributed to the stretching vibration of the O/N-H bond. Peaks observed at 2919.34 cm^−1^ and 2850.68 cm^−1^ are linked to -CH_3_ and -CH_2_ structures, respectively. Moreover, the absorption peak detected at 1643.57 cm^−1^ is associated with the C=O bond, whereas the peak at 1384.64 cm^−1^ indicates the C-N stretching vibration [17,18]. Peak located at 1175.93 cm^−1^ indicates the presence of aromatic alkoxy bonds. The results of infrared spectroscopy showed that the surface of SMC-CDs contained functional groups such as amino, carbonyl, and hydroxyl groups, which may be related to their biological activities.

**Figure 2:**
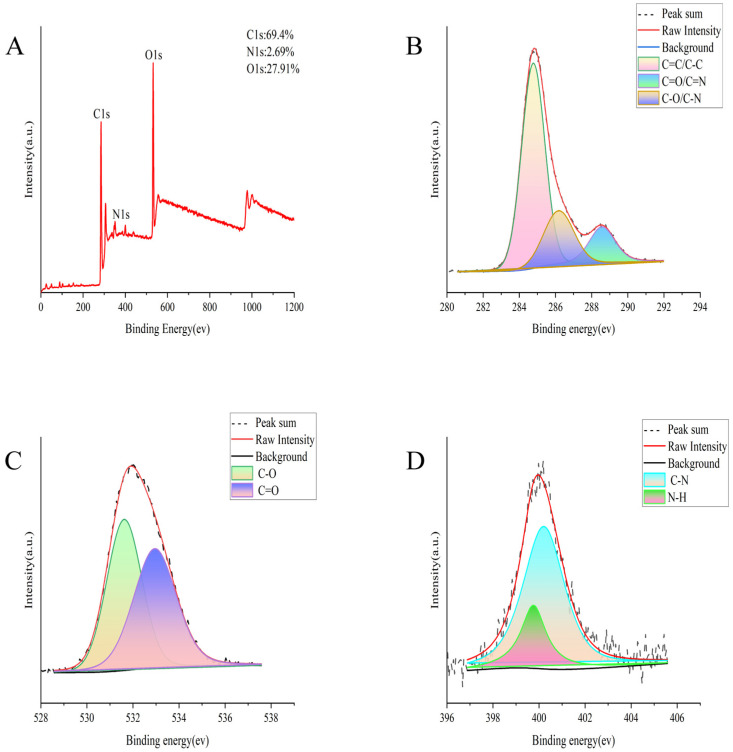
The surface composition and elemental characterization of the synthesized SMC-CDs via XPS analysis. (**A**) Full-scan x-ray photoelectron spectroscopy of SMC-CDs. (**B**) The scan mode outcomes for the C1s of SMC-CDs. (**C**) The scan mode outcomes for the O1s of SMC-CDs. (**D**) The scan mode outcomes for the N1s of SMC-CDs.

We detected the maximum excitation and emission wavelengths of SMC-CDs using fluorescence spectrometry. As shown in Figure 1H, SMC-CDs exhibit maximum excitation and emission wavelengths at 348 nm and 455 nm, respectively. XPS was utilized to analyze the elemental composition and coordination information of SMC-CDs. The composition mainly consists of elements C, O and a small amount of N. The proportions are as follows: C (69.4%), O (27.91%), and N (2.69%), respectively (Figure 2A). Based on the scan mode results of C1s, three peaks were identified around 284.75 eV, 286.16 eV, and 288.57 eV, corresponding to C=C/C-C, C-O/C-N, and C=O/C=N, respectively. The O1s scanning pattern showed two peaks at 531.61 eV and 532.96 eV, corresponding to C-O and C=O bonds, respectively. In addition, the N1s scanning pattern showed two peaks at 399.73 eV and 400.18 eV, corresponding to N-H and C-N, respectively. The XPS analysis of SMC-CDs indicates the presence of nanoscale components bearing functional groups like carboxyl, hydroxyl, and amino groups, a notion supported by the findings of the infrared spectroscopy analysis [19].

### 2.2. Effect of SMC-CDs on ISO-Induced Electrocardiogram Parameters in Rats

**Figure 3:**
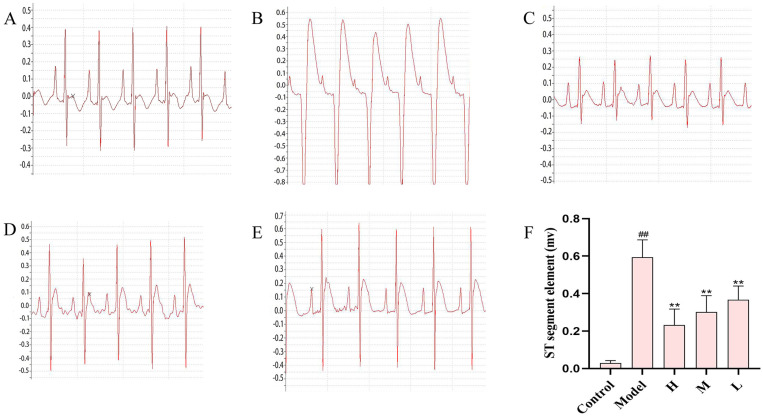
SMC-CD pretreatment reduces the severity of myocardial infarction in rat models. Electrocardiogram of the (**A**) control group, (**B**) model group, (**C**) high-dose SMC-CDs group, (**D**) medium-dose SMC-CDs group and (**E**) low-dose SMC-CDs group. (**F**) Changes in the ST segment of the ECG in each group of rats. Data are represented as the means ± SD (*n* = 5). ^##^ *p* < 0.01 vs. the control group, ** *p* < 0.01 vs. the model group.

The electrocardiogram (ECG) is the primary diagnostic tool for detecting cardiac disorders. In the event of an AMI, specific changes become apparent in the ST segment of the electrocardiogram (ECG). Furthermore, an elevation in the ST segment exceeding 0.2 mV frequently indicates the initiation of an acute infarction [20]. In Figure 3, the ST segment of the electrocardiogram in rats from the model group showed a marked increase compared to the control group (*p* < 0.01), underscoring the severity of myocardial infarction in the model group. This indicates the successful establishment of the acute myocardial infarction model. The electrocardiogram results of rats from the SMC-CD treatment groups at all dosage levels—low, medium, and high—demonstrated significant decreases compared to the model group, with the data showing substantial statistical differences (*p* < 0.01). Additionally, the reduction in ST segment levels exhibited dose-dependency, increasing in magnitude with higher doses of SMC-CDs.

### 2.3. Effect of SMC-CDs on ISO-Induced Echocardiographic Parameters in Rats

**Figure 4:**
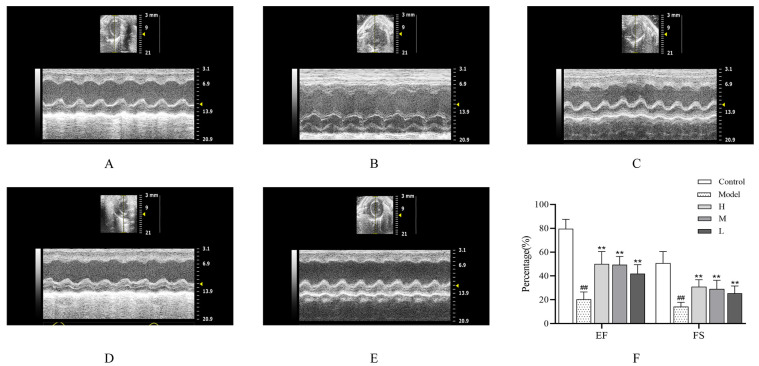
The preprocessing of SMC-CDs can significantly enhance the functional performance of rat hearts. Echocardiogram of the (**A**) control group, (**B**) model group, (**C**) high-dose SMC-CDs group, (**D**) medium-dose SMC-CDs group and (**E**) low-dose SMC-CDs group. (**F**) Assessments of left ventricular ejection fraction (EF) and fractional shortening (FS) were conducted in rats across each experimental group. Data are represented as the means ± SD (*n* = 5). ^##^ *p* < 0.01 vs. the control group, ** *p* < 0.01 vs. the model group.

Echocardiographic assessments of left ventricular ejection fraction (EF) and fractional shortening (FS) can detect lesions of various types occurring in real time. In cases of rat myocardial ischemic infarction, the EF and FS will be lower than normal value, making echocardiography a vital tool of detecting myocardial infarction in rats [21]. Compared to the control group, as illustrated in Figure 4A–F below, the EF and FS of rats in the model group showed a marked decline (*p* < 0.01); Conversely, the EF and FS of rats in the SMC-CDs groups exhibited a significant increase (*p* < 0.01), demonstrating a dose-dependent increase.

### 2.4. Histopathological Examination of Myocardial Tissue

**Figure 5:**
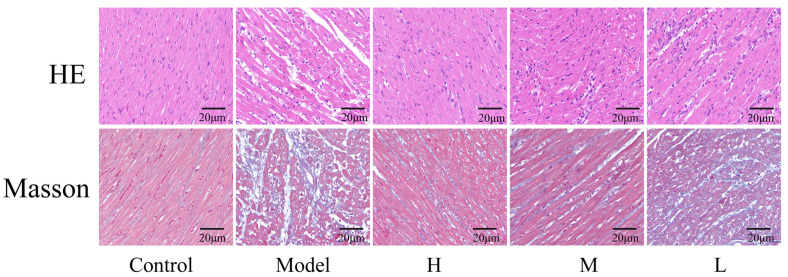
The effect of SMC-CDs on myocardial histopathology in a rat model of acute myocardial infarction. (HE, Masson ×400.)

Hematoxylin–eosin (HE) staining and Masson staining were used to observe the histopathological alterations in the myocardial tissue of rats across different groups (Figure 5). HE staining showed that cardiomyocytes and myofibrils of the control group of rats were regular in morphology and tightly arranged. There was an absence of vacuolar deformation within the sarcoplasm, and no evidence of neutrophil infiltration or congestive edema was observed. In the model group, cardiomyocytes exhibited disarray and numerous vacuoles, alongside necrosis. The interstitium of myocardium exhibited congestion and edema, showing varying degrees of cellular necrosis. The transverse striations in the myocardium were considerably disrupted, and myofibrils were fractured and lysed. After 2 weeks of SMC-CD pre-administration, all groups of rats demonstrated notable improvement in myocardial histopathological morphology. There was a significant reduction in neutrophil infiltration, and interstitial congestion and edema. Additionally, the arrangement of myofibrils showed a trend towards normalization, with the most pronounced effects observed in the high-dose group. Masson staining showed that the myocardial fibers in the control rats were tightly arranged with minimal collagen proliferation. In contrast, the rats in the model group exhibited significant collagen fiber proliferation within the cardiomyocyte interstitium, with some fibers merging into collagen connective tissue, culminating in advanced myocardial fibrosis. Pre-administration of SMC-CDs led to a marked reduction in myocardial fibrosis across all rat groups, with the most pronounced improvement observed in the high-dose cohort.

### 2.5. Effect of SMC-CDs on the Extent of ISO-Induced Myocardial Infarction in Rats

**Figure 6:**
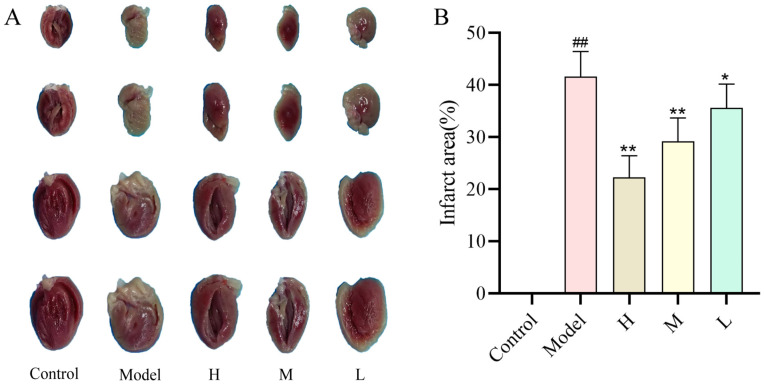
Myocardial infarction was identified in rats from each group utilizing a histological TTC staining assay. (**A**) Images of the heart from each group. (**B**) Percentage of myocardial infarct area by semi-quantification. Data are represented as the means ± SD (*n* = 5). ^##^ *p* < 0.01 vs. the control group, ** *p* < 0.01 and * *p* < 0.05 vs. the model group.

Triphenyltetrazolium chloride (TTC) staining is one of the most common stains used to detect the size of cardiac infarcts in experimental animals, and infarcted myocardial tissue can be stained white [22]. The myocardial tissue of control group rats showed no infarcted regions, resulting in exclusively red staining results (Figure 6A). In comparison to the control group, myocardial tissues from rats in the model group exhibited prominent infarcted regions, and display a significantly higher myocardial infarction rate (Figure 6B). Compared to the control group, both the myocardial infarction areas and rates in the SMC-CD pre-administered groups significantly decreased (Figure 6A,B). Statistically significant differences were found among all groups. Pre-administration of SMC-CDs over a two-week period significantly reduced the incidence of myocardial infarction in rats afflicted with acute myocardial infarction (AMI). Additionally, there was a notable reduction in the infarct area size proportional to the administered dosage. The potential cause behind this phenomenon may be attributed to the improvement of the myocardial antioxidant abilities in the rats.

### 2.6. Effect of SMC-CDs on ISO-Induced Serum Cardiac Biomarker Enzymes in Rats

**Figure 7:**
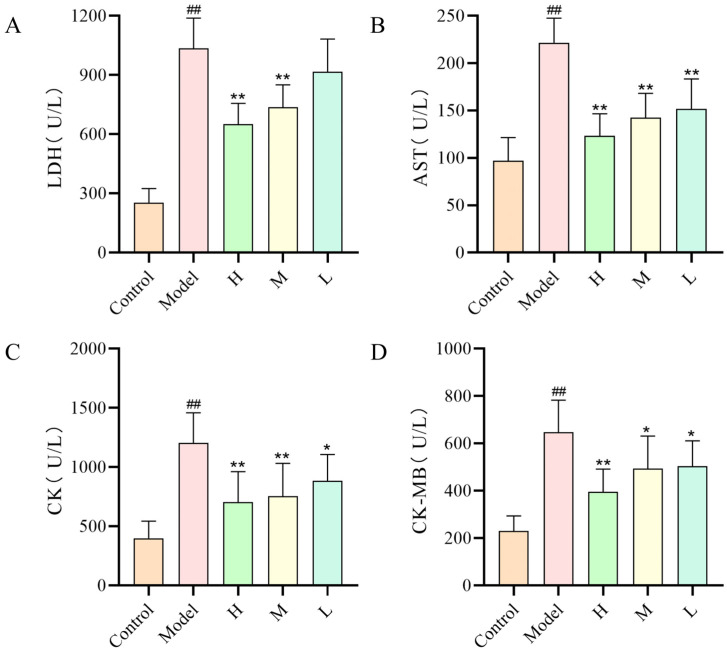
The effects of SMC-CDs on serum cardiac biomarker enzyme levels in different groups of rats. (**A**) LDH levels, (**B**) AST levels, (**C**) CK levels and (**D**) CK-MB levels in each group. Data are represented as the means ± SD (*n* = 8). ^##^ *p* < 0.01 vs. the control group, ** *p* < 0.01 and * *p* < 0.05 vs. the model group.

Serum biomarker testing is essential not only for the prompt diagnosis of acute myocardial infarction (AMI) but also for its prognostic evaluation. Serum levels of AST, LDH, CK and CK-MB serve as key indicators of myocardial injury severity, thus myocardial enzyme assays are frequently utilized as foundational diagnostic tools for AMI [23]. The accuracy of single myocardial marker enzymes in diagnosing AMI is low, while the combination of multiple indicators can improve diagnostic accuracy, so the clinic often combines multiple myocardial marker enzymes for more accurate diagnosis of AMI. As illustrated in Figure 7A–D, serum concentrations of cardiac-specific marker enzymes exhibited a significant elevation in the model group of rats when compared to the control group (*p* < 0.01). In contrast, rats administered SMC-CDs (at doses of 1.5, 3, and 6 mg/kg, respectively) successfully reduced myocardial enzyme levels relative to the model group, indicating a cardioprotective effect.

### 2.7. Effect of SMC-CDs on ISO-Induced Myocardial Antioxidant Activity and Na^+^-K^+^-ATPase and Ca^2+^-Mg^2+^-ATPase Activities in Rats

ATPase is present within in tissue cells and organelles and is a protein in biological membranes responsible for energy conversion and information transfer [24]. Na^+^, K^+^-ATPase and Ca^2+^, Mg^2+^-ATPases are essential for regulating Ca^2+^ levels within cells to preserve homeostasis. When their activity decreases, this can result in an increase in intracellular Ca^2+^ concentration, which in turn leads to an increase in myocardial contractility and oxygen demand, ultimately inducing apoptotic necrosis of cardiomyocytes. Therefore, the level of Na^+^, K^+^-ATPase and Ca^2+^, Mg^2+^-ATPase content in myocardial tissues can serve as an indirect indicator of the extent of damage to cardiomyocytes. Oxidative stress is a crucial factor in the initiation of AMI, and repeated use of ISO in high doses induces oxidative stress in cardiomyocytes, which in turn leads to myocardial ischemic infarction [25]. SOD, GSH and CAT are essential antioxidant enzymes in the body, which can reduce H_2_O_2_ produced during oxidation reactions in the body to H_2_O, thereby preventing the damage to oxygen free radicals and H_2_O_2_ to the organism’s tissue cells. MDA, which acts as a biomarker for oxidative stress induced by the peroxidation of polyunsaturated fatty acids, frequently accumulates in the body during myocardial ischemic infarction, thereby aggravating myocardial damage.

**Figure 8:**
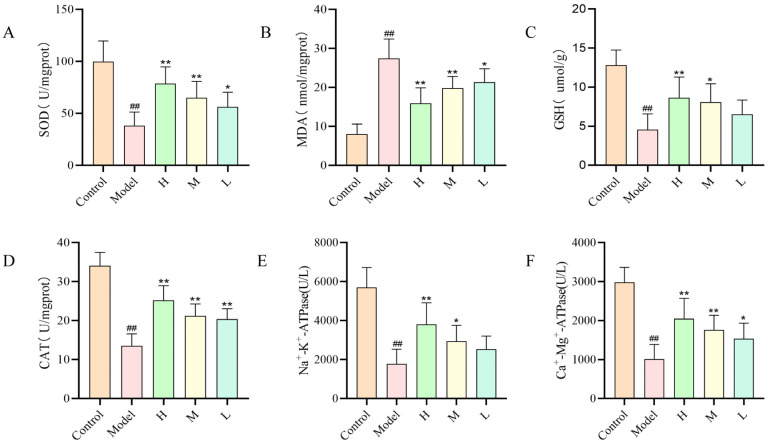
The effect of SMC-CDs on myocardial antioxidant activity (**A**–**D**) and the activities of ATPase in rats. (**E**,**F**) in myocardial tissues from various rat groups. (**A**) SOD levels, (**B**) MDA levels, (**C**) GSH levels and (**D**) CAT levels in each group. (**E**) Na^+^-K^+^-ATPase activity and (**F**) Ca^2+^-Mg^2+^-ATPase activity in each group. Data are represented as the means ± SD (*n* = 8). ^##^ *p* < 0.01 vs. the control group, ** *p* < 0.01 and * *p* < 0.05 vs. the model group.

The experimental results are presented in Figure 8. Compared to the control group, the antioxidant enzyme activities as well as the related ATPase activities of myocardial tissues were greatly decreased in the model group of rats. Conversely, in the SMC-CD pre-administered groups (1.5, 3 and 6 mg/kg, respectively) a significant increase in ATPase activity and antioxidant enzyme activity was observed, indicating cardioprotective effects in rat cardiomyocytes.

### 2.8. Effect of SMC-CDs on ISO-Induced Apoptosis in Rat Cardiomyocytes

**Figure 9:**
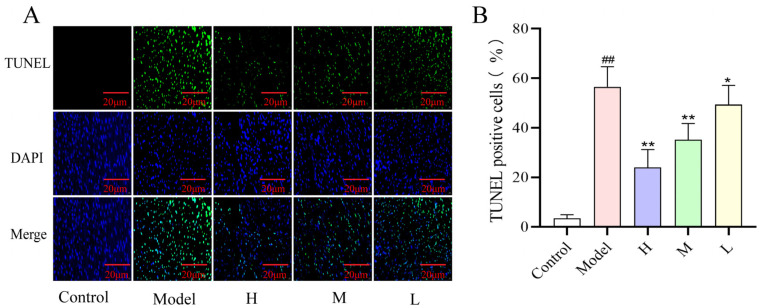
The effect of SMC-CDs on cardiomyocyte apoptosis within rat myocardial tissue. (**A**) The results of the TUNEL assay for each group (magnification = 400×). (**B**) Percentage of TUNEL-positive cells. Data are represented as the means ± SD (*n* = 5). ^##^ *p* < 0.01 vs. the control group, ** *p* < 0.01 and * *p* < 0.05 vs. the model group.

The TUNEL assay is a highly sensitive and rapid technique for the detection of apoptosis. The nuclei of normal cells show a clear blue fluorescence under a fluorescence microscope after relevant staining, while the nuclei of positive apoptotic cells show a green fluorescence [26]. As shown in below, a minimal presence of apoptotic cells was detected in the myocardial tissues of rats belonging to the control group (Figure 9A). In contrast, a significant accumulation of green fluorescently labeled apoptotic cells was observable under the microscope in the myocardial tissues of rats from the ISO-induced model group (Figure 9B), with the apoptotic rate being notably different from that of the control group. Pre-administration of SMC-CDs for two weeks (Figure 9A,B) markedly reduced cardiomyocyte apoptosis, with a notably lower apoptotic rate observed in comparison to the model group. Furthermore, the rate of cardiomyocyte apoptosis in each of the SMC-CDs dosing groups was correlated with the dosage administered, decreasing progressively as the dosage increased. The findings of this study suggest that SMC-CDs may provide cardioprotective effects by inhibiting cardiomyocyte apoptosis.

### 2.9. Effect of SMC-CDs on In Vitro Cell Viability

**Figure 10:**
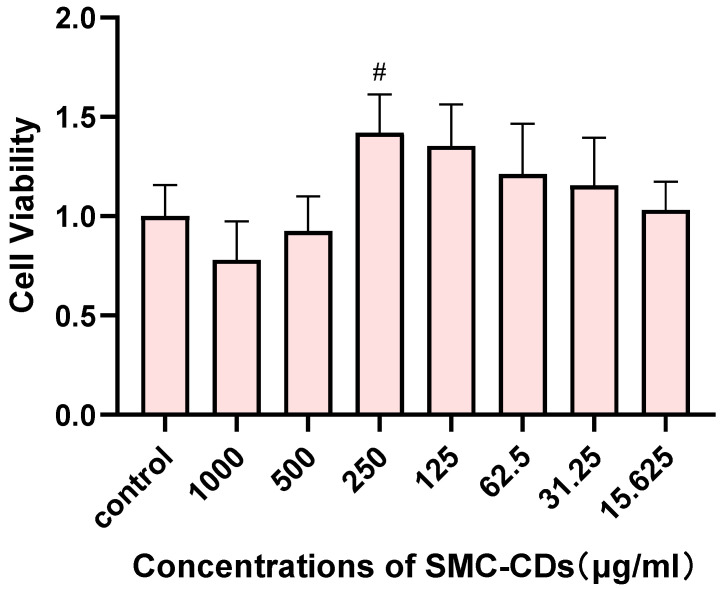
Cell viability of H9c2 cells after incubation with various concentrations of SMC-CDs for 24 h. ^#^ *p* < 0.05 vs. the control group.

Figure 10 illustrates the effects of SMC-CDs on the viability of H9c2 cells across seven different concentrations. The results indicate that within the concentration range of 15.625 to 250 μg/mL of SMC-CDs, cell viability improved as the concentration increased, with the 250 μg/mL concentration exhibiting a notably significant pro-enhancement effect (*p* < 0.05). However, upon elevating the concentration of SMC-CDs from 500 to 1000 μg/mL, a marginal decrease in H9c2 cell viability is observed (*p* > 0.05). These results indicate negligible in vitro cytotoxicity at SMC-CDs concentrations of 15.625~1000 μg/mL.

## 3. Discussion

AMI is a common clinical acute ischemic heart disease, the incidence of which is rising year by year. When an AMI occurs, the patient will feel severe crushing pain in the precordial area or behind the sternum, and the disease has a rapidly onset and a vicious condition, seriously threatening human life and health [27]. This condition is acute ischemia causing myocardial necrosis, followed by an endogenous inflammatory response that further leads to myocardial injury and dysfunction [28]. Apoptosis is the predominant manifestation of ischemic injury in cardiomyocytes. The mechanism of action is characterized by a reduction in oxygen levels during ischemic damage to myocardial cells, which in turn disrupts the cellular oxidative respiratory chain and the free radical scavenging system within the body, leading to the accumulation of a large number of free radicals. This accumulation alters the structural integrity of the cell membrane, ultimately leading to damage to myocardial cells and potentially progressing to apoptosis [29]. An increasing body of research indicates that oxidative stress and apoptosis are the main drivers of AMI occurrence and development [30,31,32]. In this study, we comprehensively analyzed the role of SMC-CDs in AMI injury and further explored their protective effects and potential mechanisms.

Most of the treatment strategies for AMI are timely reperfusion of the ischemic area, such as pharmacological thrombolytic therapy and percutaneous coronary intervention, which can improve the symptoms and prognosis of AMI patients to some extent [33]. However, the restoration of coronary blood flow reconstruction can cause reperfusion injury of myocardial tissue and eventually lead to further expansion of myocardial infarction area [34]. Therefore, there is a need to find a new alternative or complementary therapy to decrease the incidence of myocardial infarction and to enhance clinical symptoms after infarction. Traditional Chinese medicine is widely employed in the treatment of cardiovascular diseases due to its eco-friendly safety profile and remarkable effectiveness [35]. Modern research has shown that Chinese herbs can protect the heart from ischemic injury by reducing myocardial oxygen consumption, scavenging free radicals, inhibiting inflammatory responses, improving mitochondrial function of cardiomyocytes, and other multi-pathways and multi-targets [36].

Nanotechnology has accumulated extensive research related to treating cardiovascular diseases. Nanotechnology-based cardiac therapies possess diverse therapeutic capabilities, including anti-inflammatory [37], antioxidant [38], and anti-apoptotic [39] effects. Carbon dots (CDs), a new member of the carbon family, have attracted much interest because of their unique properties. Charcoal medicine is one of the most distinctive traditional medicines in Chinese medicine, which has been used for more than 2000 years since the earliest documentation of charcoal medicine. Charcoal is a method of preparing Chinese medicine by frying raw herbs over a hot flame until the surface is charred black and the interior is charred yellow. High-temperature carbonization is an important part of the carbon preparation process, which resembles the process of preparing carbon dots by “high-temperature pyrolysis”. Through extensive research, our team has discovered that charcoal medicines contain carbon dots, similar in size and structure to manufactured carbon dots, and confirmed their broad biological capabilities [40]. *Salvia miltiorrhiza*, an ancient medicinal plant renowned for its use in treating heart conditions, is distinguished by its exceptional strength, sustainability, and cost efficiency. Frequently subjected to a method of thermal decomposition, it can amplify its healing properties while reducing adverse reactions [41]. Building on its historical use in traditional Chinese medicine and our preliminary study, we synthesized SMC-CDs with a particle size of less than 10 nm employing a simple and environmentally friendly heating process. Furthermore, we demonstrated that SMC-CDs exhibit non-toxicity to cells at concentrations ranging from 15.625 to 250 μg/mL and enhance cell viability, as evidenced by the CCK-8 assay, thereby establishing SMC-CDs as a novel and safe therapeutic compound.

The most widely used clinical technique for diagnosing of cardiac conditions is the electrocardiogram. Observable changes in the ST segment of the ECG are indicative of an AMI occurrence. When the ST segment of the electrocardiogram is elevated by more than 0.2 mv, it often represents the occurrence of acute infarction, so the extent of myocardial infarction can be assessed based on the degree of ST segment deviation [42]. An essential tool for determining cardiac function is echocardiography. In echocardiography, ejection fraction (EF) and shortening fraction (FS) are frequently utilized as vital parameters to evaluate cardiac function, and when myocardial ischemia occurs, the EF and FS values will be lower than normal, so echocardiography is often used clinically as an important adjunct to the electrocardiogram to assess cardiac function [21]. In the present study, the ISO-induced model group of rats were observed a marked increase in the ST segment of the electrocardiogram (>0.2 mv) and a significant decrease in EF and FS. While administering SMC-CDs as a pretreatment two weeks significantly decreased ST segment elevation on the electrocardiogram and notably raised EF and FS values. This indicates that SMC-CDs exert a positive inotropic effect, thereby mitigating myocardial damage induced by ISO.

At the early stage of AMI, most patients do not have typical clinical symptoms and specific changes cannot be detected by electrocardiogram, so serum biochemistry is crucial to the early diagnosis of AMI. CK is an enzyme that facilitates the reversible transformation of creatine and ATP into phosphocreatine and ADP [43]. This reaction predominantly generates ATP under neutral pH conditions, thereby sustaining the energy requirements of tissue cells. CK-MB is one of the three isoforms of creatine kinase, with approximately 20% of the total creatine kinase present in the myocardium existing as the MB isozyme. The concentrations of total CK and CK-MB are directly associated with the extent of myocardial infarction, serving as significant prognostic indicators [44]. LDH is a crucial enzyme involved in energy metabolism, catalyzing the conversion of pyruvate to lactate, and plays a vital role in both glycolysis and gluconeogenesis [45,46]. However, following a myocardial infarction, there is a reduction in myocardial energy levels alongside an elevation in glycolytic activity and lactate production. AST is an enzyme located in the mitochondria and cytoplasm, with its elevation level being dependent on the duration of myocardial injury. When myocardium undergoes ischemic infarction, myocardial cell membranes are damaged and myocardial enzyme release into the bloodstream, including serum levels of CK, CK-MB, LDH, and AST, correlating with the degree of ischemic myocardial injury [43,47]. Damage to myocardial cells is primarily caused by inadequate energy supply, and excessive myocardial contraction resulting from high intracellular Ca^2+^ levels, which leads to increased oxygen demand in cardiomyocytes and degradation of the associated ATPases, and ultimately to cardiomyocyte injury. Therefore, the degree of myocardial tissue damage can be assessed by detecting the level content of the relevant ATPase in myocardial tissue [48]. The present study suggests that SMC-CDs could potentially demonstrate cardioprotective benefits by reducing the serum concentrations of cardiac enzymes in the rat model group and enhancing the functions of associated ATPases in myocardial tissues.

One of the main factors in the pathophysiology of AMI is oxidative stress. High-dose use of ISO causes cardiomyocytes to produce a large amount of ROS [49], which triggers lipid peroxidation by attacking polyunsaturated fatty acids in the biofilm and produces lipid peroxides, such as MDA and LDL. These products cause excessive oxidative stress within the myocardial tissues, which leads to a series of reactions, such as loss of cellular membranes, mitochondrial disruption, and degradation of ATPases and ultimately leads to apoptosis and necrosis of cardiomyocytes. Scavenging enzymes like SOD, GSH, and CAT are the first line of defense in the antioxidant system; they remove excess reactive oxygen species produced by the organism’s oxidative processes [50]. Reduced activity of SOD, which is typically present in the plasma membrane, is a key indicator of oxidative stress. GSH lowers lipid peroxidation and hydrogen peroxide, shielding cell membranes from oxidative damage. Additionally, the CAT, a tetrameric hemoglobin, facilitates the elimination of hydrogen peroxide. MDA as a marker produced by peroxidation of polyunsaturated fatty acids, and low oxygen levels and oxidative stress induced by acute ischemic injury can lead to MDA accumulation in the myocardium. The findings of the current research showed that high dose of ISO caused excessive oxidative stress in myocardial tissues of AMI rats, whereas SMC-CD pre-administration could protect ischemic cardiomyocytes by counteracting excessive oxidative stress induced by free radicals by increasing the levels of SOD, CAT, and GSH, and decreasing the level of MDA in the cardiac tissues. A growing body of research indicates that oxidative stress activates triggers various cellular signaling pathways, including apoptotic pathways. TUNEL assay results from myocardial tissues demonstrated that SMC-CDs effectively inhibited the apoptotic of cardiomyocytes in rats with acute myocardial infarction, which corresponded to its antioxidant activity. For example, Strychnine regulates oxidative stress and apoptosis by activating JAK2/STAT3 signaling pathway, thereby preventing myocardial ischemia–reperfusion injury [51]. Furthermore, Ginsenoside Rc stimulates the SIRT1 signaling pathway, deacetylates Fox01, reduces mitochondrial oxidative stress and inhibits apoptosis [52]. These findings indicate that SMC-CDs exert their specific biological effects by attenuating oxidative stress and inhibiting apoptosis, ultimately protecting cardiac function.

In conclusion, SMC-CDs demonstrate preventive protection effect against acute ischemic myocardial injury by reducing myocardial infarction area, decreasing cardiac myosin activity, and increasing free radical scavenging enzyme activity to inhibit apoptosis.

## 4. Materials and Methods

### 4.1. Chemicals

*Salvia miltiorrhiza* Carbonisata was procured from Beijing Qian Cao Herbal Pieces Co., Ltd. (Beijing, China). Isoproterenol hydrochloride was obtained from Bairdi Biotechnology Co., Ltd., both companies located in Beijing, China. Propranolol hydrochloride was sourced from Beijing Zheng Cheng Biotechnology Co., Ltd. (Beijing, China). Na^+^/K^+^-ATPase, Ca^2+^/Mg^2+^-ATPase ELISA kits were purchased from SapoRex Co., Ltd. (Beijing, China). Dialysis membranes of 1000 Da molecular weight cut off were acquired from Beijing Ruida Henghui Technology Development Co., Ltd., Beijing, China. The enzymes superoxide dismutase (SOD), glutathione (GSH), malondialdehyde (MDA), and catalase (CAT) were procured from the Nanjing Jiancheng Bioengineering Institute located in Nanjing, China. All experiments were performed utilizing deionized water.

### 4.2. Preparation and Characterization of SMC-CDs

#### 4.2.1. Preparation of SMC-CDs

**Figure 11:**
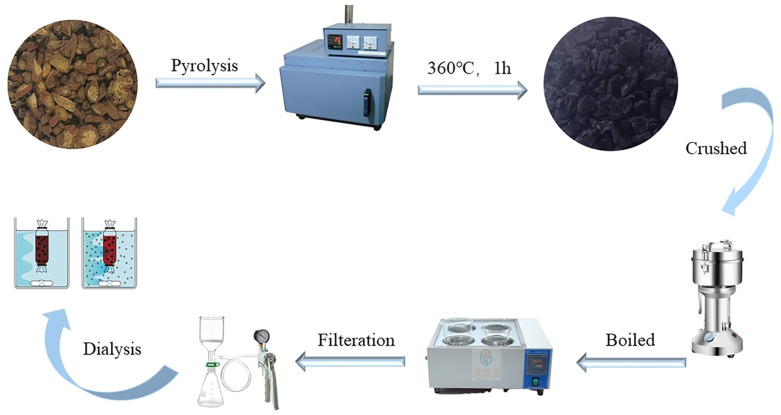
The schematic diagram detailing the synthesis procedure of SMC-CDs.

As per previous reports, *Salvia miltiorrhiza* Carbonisata (SMC) was used as the carbon source in a one-step, high-temperature pyrolysis process to create SMC-CDs. First, 400 g of SMC were placed in crucibles, the tops secured, and sealed with foil paper to ensure a tight fit. Second, the crucibles were calcined for one hour at 360 °C in a muffle furnace to produce SMC. Then, SMC was ground using a micro pulverizer once the temperature dropped to room temperature. The obtained particles of homogeneous crushed drug were boiled in a water bath at 100 °C for 2 times for 1 h each time. To obtain the SMC-CDs, the final solution underwent dialysis against distilled water for seven days using a 1000 Da membrane following filtration through a 0.22 μm cellulose acetate membrane. Figure 11 shows the schematic diagram of the SMC-preparation experimental protocol.

#### 4.2.2. Characterization of SMC-CDs

The microstructure, particle size distribution, and morphology of the SMC-CDs were analyzed utilizing a TEM (Tecnai G220; FEI Company, Hillsboro, OR, USA) under an accelerating voltage of 100 kV. The Structural properties and atomic lattice fringes were investigated using HRTEM (JEN-1230; Japan Electron Optics Laboratory, Mitaka, Japan). FTIR (Fourier transform infrared) spectroscopy (Thermo, Carlsbad, CA, USA) was employed to analyze the functional group composition on the surface of SMC-CDs across the spectral range of 400 to 4000 cm^−1^. The UV-Vis spectrum and fluorescence characteristics were measured and analyzed with UV-Vis spectroscopy (CECIL, Cambridge, UK) and fluorescence (F-4500, Tokyo, Japan) spectroscopy instruments, respectively. Cu K-alpha radiation was utilized for X-ray diffractography analysis (D8-Advanced X-ray diffractometer, Bruker AXS, Karlsruhe, Germany). X-ray photoelectron spectroscopy (XPS) was employed to analyze the surface composition of SMC-CDs using an ESCALAB 250Xi spectrometer (Thermo Fisher Scientific, Waltham, MA, USA) equipped with a monochromatic AlK X-ray source operating at 150 W.

### 4.3. Animals and Experimental Protocols

#### 4.3.1. Animals

This study utilized SPF-grade healthy male Sprague Dawley rats averaging 220 g from Beijing SiPeiFu Biotechnology Co., Ltd. (Beijing, China). Kept in standard conditions (24.0 ± 1.0 °C, 55–65% humidity) at the Experimental Animal Center of Beijing University of Chinese Medicine, they followed a regular light/dark cycle with free access to food and water. The protocol received approval from the Animal Experimentation Ethics Committee at Beijing University of Chinese Medicine (BUCM) and adheres to the Health Guidelines for the Care and Use of Laboratory Animals.

#### 4.3.2. Experimental Procedure

Sprague Dawley (SD) rats were divided into five groups of eight. Group I, designated as the control group (Control), was administered saline via intragastric route for a duration of 14 days. Groups II designated as ISO-induced AMI group (Model), was administered saline via intragastric route for a duration of 14 days, followed by multiple subcutaneous injections of ISO (85 mg/kg) [53] over a two-day span on the 13th and 14th days. Groups III, IV, and V were designated as low, medium, and high doses of SMC-CDs combined with ISO, respectively. Specifically, they were administered 1.5 mg/kg, 3 mg/kg, and 6 mg/kg of SMC-CDs daily via intragastric route for 14 consecutive days, with subcutaneous injections of ISO (85 mg/kg) administered multiple times on days 13 and 14. A prominent ST segment elevation on the ECG indicates successful modeling.

### 4.4. Echocardiography

Twelve hours after the completion of second modeling, rats in each group were given a subcutaneous injection of 10% chloral hydrate (300 mg/kg) to induce anesthesia. The cardiac performance of rats was assessed using echocardiography via a Vevo2100 Ultrasound system, which was outfitted with a 10 MHz linear array ultrasound transducer. Measurements were taken separately for the left ventricular end-systolic internal diameter (LVESD) and the left ventricular end-diastolic internal diameter (LVEDD). Subsequently, these measurements were used to calculate left ventricular ejection fraction (EF) and fractional shortening (FS) to provide a comprehensive assessment of LV systolic function.

### 4.5. Histological Examination

Following the physicochemical index tests conducted on each group of rats, anesthesia was administered, the abdominal aorta was punctured, and blood samples were extracted, and the rats were executed. These heart tissues were immediately extracted postmortem from the rats and preserved in a 10% formalin solution. And then they were subjected to gradient dehydration, paraffin embedding, sectioning, and staining with Masson’s trichrome, hematoxylin, and eosin (HE) stains. Subsequently, the pathological changes in each group of cardiac tissues were observed using a light microscope (Nikon, Tokyo, Japan).

### 4.6. Tissue Necrosis Assessment

The triphenyltetrazolium chlorinated (TTC) Evans blue tissue enzyme staining method was employed for assessing tissue necrosis. Removed hearts were placed on ice and cut into 5 slices perpendicular to the line between the apex and the base of the heart. Each slice was incubated in a 37 °C water bath containing 1% TTC buffer for 15 min in the dark, and then removed. Greyish-white is the infarcted region (IS), while red is the non-infarcted region (NIS).

Percentage of infarction = area of infarcted area/area of left ventricle × 100%.

### 4.7. Serum Biochemical Analysis

Following anesthesia, blood was drawn from the abdominal aorta of the rats, centrifuged at a speed of 3500 rpm for 10 min at 4 °C to obtain serum. Then, an automatic biochemical analyzer was used to measure the serum levels of lactate dehydrogenase (LDH), aspartate aminotransferase (AST), creatine kinase (CK), and creatine kinase isoenzyme (CK-MB).

### 4.8. Assessment of Oxidative Stress and ATPase Activities in Myocardial Tissue

After homogenizing the heart tissue with saline, the mixture was centrifuged at 3000 rpm for 10 min at 4 °C to collect the supernatant, which was then used to make the 10% heart homogenate. Relevant ATPase activities and oxidative stress levels in cardiac tissues were measured using commercially available ELISA kits, including indicators of Na^+^-K^+^-ATPase, Ca^2+^-Mg^2+^-ATPase, SOD, GSH, MDA and CAT. The measurement of all indices was performed with a microplate reader, following the instructions provided by the manufacturer for the assay kit.

### 4.9. TUNEL Assay

Myocardial tissues were fixed using 4% paraformaldehyde and subsequently rinsed thoroughly with double-distilled water. Then, it was paraffin-embedded and cut into 2–3 micron thick slices, placed in TUNEL assay reagent solution and incubated for 60 min at room temperature. Subsequently, an appropriate amount of DAPI staining solution was added for staining, and after the staining was completed, anti-fluorescence quencher was added to seal the slices, and finally observed under the optical high magnification microscope. Cells from random 5 fields of view were selected to evaluate the apoptosis rate, which was calculated as: apoptosis rate = number of apoptotic cells/total number of cells × 100%.

### 4.10. Cell Viability Assay

The cytotoxicity of SMC-CDs on H9c2 cells were assessed utilizing the CCK-8 assay. The cells were cultured in DMEM medium supplemented with 20% FBS and maintained in a 5% CO_2_ incubator at 37 °C. Following cell counting, they were plated into a 96-well plate at a density of 1 × 10^5^ cells per well, with 100 μL of medium added to each well. Blank, control, and experimental groups were established for comparison. After a 24 h incubation period, the blank group received 100 μL of DMEM medium containing 20% FBS in the designated wells, while the control group was administered 100 μL of the same medium in the cell wells. The experimental group was treated with 100 μL of SMC-CDs solutions at varying concentrations of 1000, 500, 250, 125, 62.5, 31.25, and 15.625 μg/mL, with six replicates for each concentration, followed by an additional 24 h incubation. Post-incubation, the cells were washed three times with PBS solution, and 100 μL of CCK-8 working solution was introduced to each well, followed by a further 3 h of incubation. Ultimately, the optical density (OD) of each well was quantified at a wavelength of 450 nm using a microplate reader. Cell viability was determined using the formula:

Cell viability (% relative to control) = ((experimental group OD − blank group OD)/(control group OD − blank group OD)) × 100%.

In this formula, the OD values represent the absorbance at 450 nm for the experimental, blank, and control groups, respectively.

### 4.11. Statistical Analysis

IBM SPSS Statistics (version 26) was utilized to conduct statistical analyses for every experiment. Data with a normal distribution and homogeneity of variance were given as the mean ± standard deviation (SD). To compare the differences between the groups, one-way analysis of variance (ANOVA) was used. The Kruskal–Wallis test and a post hoc test were used to analyze non-normally distributed data using non-parametric statistics; differences between *p* < 0.05 and *p* < 0.01 were regarded as statistically significant.

## 5. Conclusions

In this research, SMC-CDs were for the first time efficiently isolated from the natural herb *Salvia miltiorrhiza*. The findings demonstrated that pretreatment with SMC-CDs conferred significant protective effects on rat cardiomyocytes and markedly enhanced cardiac function in rats with acute myocardial infarction (AMI). The underlying mechanism is likely associated with the augmentation of ATPase activity and the antioxidative capacity of myocardial tissues, which play a essential role in inhibiting cardiomyocyte apoptosis, thus safeguarding myocardial integrity. From a nanomedicine perspective, SMC-CDs offer a novel therapeutic avenue for addressing acute myocardial infarction, instilling renewed hope for treatment strategies.

## Data Availability

The data supporting this study′s findings are available from the corresponding author upon reasonable request.
